# Divergent Metabolic Regulation of Autophagy and mTORC1—Early Events in Alzheimer’s Disease?

**DOI:** 10.3389/fnagi.2017.00173

**Published:** 2017-06-02

**Authors:** Mai A. Shafei, Matthew Harris, Myra E. Conway

**Affiliations:** Department of Applied Science, The University of the West of EnglandBristol, United Kingdom

**Keywords:** autophagy, mTORC1, Alzheimer’s disease, insulin, leucine, BCAT, GDH

## Abstract

Alzheimer’s disease (AD) is a progressive disease associated with the production and deposition of amyloid β-peptide (Aβ) aggregates and neurofibrillary tangles, which lead to synaptic and neuronal damage. Reduced autophagic flux has been widely associated with the accumulation of autophagic vacuoles (AV), which has been proposed to contribute to aggregate build-up observed in AD. As such, targeting autophagy regulation has received wide review, where an understanding as to how this mechanism can be controlled will be important to neuronal health. The mammalian target of rapamycin complex 1 (mTORC1), which was found to be hyperactive in AD brain, regulates autophagy and is considered to be mechanistically important to aberrant autophagy in AD. Hormones and nutrients such as insulin and leucine, respectively, positively regulate mTORC1 activation and are largely considered to inhibit autophagy. However, in AD brain there is a dysregulation of nutrient metabolism, linked to insulin resistance, where a role for insulin treatment to improve cognition has been proposed. Recent studies have highlighted that mitochondrial proteins such as glutamate dehydrogenase and the human branched chain aminotransferase protein, through metabolism of leucine and glutamate, differentially regulate mTORC1 and autophagy. As the levels of the hBCAT proteins are significantly increased in AD brain relative to aged-matched controls, we discuss how these metabolic pathways offer new potential therapeutic targets. In this review article, we highlight the core regulation of autophagy through mTORC1, focusing on how insulin and leucine will be important to consider in particular with respect to our understanding of nutrient load and AD pathogenesis.

## Introduction

**Figure 1:**
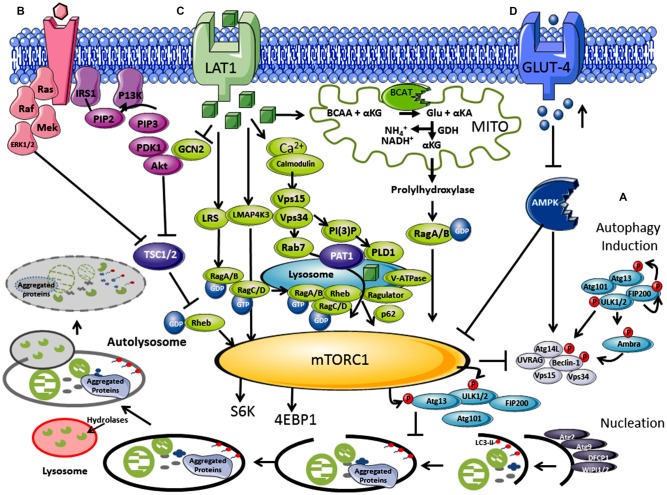
Regulation of autophagy by nutrients and hormones via the mammalian target of rapamycin (mTOR) signaling pathway. **(A)** Autophagosome *induction* begins with the activation of the ULK1/2 protein kinase complex that includes the autophagy related proteins (Atgs, where Atg13 is indispensable) and the 200 kDa focal adhesion kinase family-interacting protein (FIP200). This is followed by a *nucleation step* that is dependent on the class III phosphatidylinositol 3-kinase or human vacuolar protein sorting 34 (hVps34), complexed with BCL-2 interacting moesin-like coiled-coil protein 1 (Beclin 1) and Vps15. Phosphorylation of phosphatidylinositol (PtdIns) by hVps34 signals the recruitment of other autophagy proteins required for *elongation*. Beclin 1 is involved in two recruitment complexes, the Atg14L and UV radiation resistance-associated gene (UVRAG) complex, which are required for phagophore formation and phagocytosis, respectively. *Elongation and closure* of the autophagosome requires several Atg proteins, hVps34 and microtubule-associated light chain 3 phosphatidylethanolamine (LC3), which is regulated by GTPase Rab5. The outer membrane of the autophagosome then fuses with a lysosome, exposing the inner single membrane to lysosomal hydrolases whereby the contents are degraded. **(B)** Hormones and growth factors such as insulin and insulin-like growth factor (IGF-1) trigger mTORC1 activity through a cascade of events resulting in recruitment and activation of Akt which induces phosphorylation and degradation of tuberous sclerosis complex protein 2 (TSC2). TSC2 degradation permits GTP-bound Rheb to directly interact and activate mTORC1. The Ras-ERK pathway also activates mTORC1 through inhibitions of TSC1 and TSC2. **(C)** Amino acids, in particular leucine, regulate the mTORC1 through the Rag complex (Ras-related GTPase), which recruits mTOR to the lysosomes with Rheb and hVps34. **(D)** Inhibition of the amp-activated protein kinase (AMPK) signaling pathway is triggered by high glucose levels, resulting in the decrease of AMP:ATP ratio that inhibits TSC1 and TSC2, activating mTORC1 and inhibiting autophagy.

Alzheimer’s disease (AD), similar to other neurodegenerative diseases, is characterized by the accumulation of protein aggregates, namely amyloid β-peptide (Aβ) and Tau tangles, which lead to synaptic and neuronal damage, particularly in the hippocampal and the inferior parietal lobule (IPL) regions of the brain, resulting in memory loss (Braak and Braak, 1991; Borlikova et al., 2013). Autophagy, of which there are three types, microautophagy, chaperone-mediated autophagy and macroautophagy, is important for aggregate clearance and is considered to be dysregulated in neurodegenerative conditions, such as AD. Macroautophagy (referred to as autophagy in this review article) is the major degradation pathway in which constitutive autophagy clears functionally redundant or damaged intracellular structures whilst induced autophagy is initiated in response to environmental factors such as nutrient starvation and oxidative stress, generating recycled amino acids, lipids and other nutrients (Heras-Sandoval et al., 2014). Initially, organelles and proteins to be degraded are surrounded by an isolation membrane (phagophore) which fuses together to form a double membrane vesicle (autophagosomes; Dunn, 1990; Klionsky and Ohsumi, 1999; Figure 1A). Hydrolytic enzymes are acquired by merging of the autophagosome with acidified lysosomes (autolysosome; Appelqvist et al., 2013). Autophagosomes are then trafficked in a dynein-dependent retrograde manner along microtubules to lysosomes, which fuse to form the autolysosome (Seaman, 2012; Small and Petsko, 2015). Maturation or late-stage autophagy is fundamental to clearance and inhibition of maturation, fusion or lysosomal function can interfere with autophagic flux (Yang and Klionsky, 2010). The autophagy pathway is regulated by several signaling cascades; in particular the mammalian target of rapamycin (mTOR) pathway, which controls the initiation stage of autophagy and negatively regulates the biogenesis of lysosomes. Here, we review the role of autophagy in AD and discuss how our current understanding of nutrient load and insulin regulation are involved in its dysregulation through the mTOR pathway.

## Aberrant Autophagy in Alzheimer’s Disease

In post-mortem AD brain, autophagic vacuoles (AV) were found to have accumulated and the number of dystrophic neurites containing these AV were considerably greater relative to matched controls (Cataldo et al., 1997; Nixon et al., 2005; Nixon, 2007). High levels of Aβ and γ-secretase subunits found in AVs indicated that amyloid precursor protein (APP) processing can occur, where impaired clearance could contribute to elevated Aβ levels in the brain (Yu et al., 2005). Several studies have suggested that Aβ deposition occurs later in the disease process (Yang et al., 1998; Cataldo et al., 2000). As autophagosome imbalance is thought to occur as an early event in the pathogenesis of AD, dysregulation of this pathway may be upstream of aggregate accumulation (Perez et al., 2015). Autophagy is a multistep process (Figure 1), where a dysfunction in the formation or clearance of the autophagosome or its regulation could result in aggregate accumulation. What is clear is that the autophagy related proteins (Atg) are fundamentally important as knockout of Atg7 in mice results in neurodegenerative disease, with accumulation of ubiquitinated protein aggregates (Komatsu et al., 2005). Several other aspects of the pathway are also vulnerable and are thus seen as potential therapeutic targets. Proteins important for elongation and closure including LC3 and Beclin 1, were found to be downregulated in the IPL of AD tissue, a deficiency of which would compromise autophagosome formation (Pickford et al., 2008; Rohn et al., 2011). In APP transgenic mice models, depletion of Beclin 1 resulted in the accumulation of intracellular and extracellular Aβ, highlighting the importance of early autophagosome formation in Aβ clearance (Pickford et al., 2008). There are also indicators that end-stage processing at the autophagic/lysosomal stage is disrupted, where in AD brain, the lysosomal protease cathepsin D (intracellular aspartyl protease) was found to be upregulated (Cataldo et al., 1995). Cathepsins have β and γ secretase activity, are capable of cleaving APP, and if inhibited or deleted result in a build-up of Aβ (Mueller-Steiner et al., 2006) and tau aggregates (Hamano et al., 2008). The final stage of vesicular trafficking has also been shown to be perturbed, resulting in inefficient clearance of AVs, reducing autophagic flux (for review see Small and Petsko, 2015). Therefore it is clear that autophagy dysregulation (at all stages) has been implicated in aggregate accumulation or ineffective clearance. However, what is not clear are the mechanistic details underpinning or regulating these alterations in autophagy or how specifically it results in Aβ and tau aggreation and more so if the process begins upstream of autophagy.

## mTOR and Nutrient Modulation of Autophagy

The mTOR pathway acts as an environmental sensor, which positively regulates protein synthesis and represses autophagy. mTOR forms complexes with several different core proteins, collectively described as mTORC1 and mTORC2 (not a direct autophagy regulator; Tan and Miyamoto, 2016). Knowledge of external stimuli that regulate the P13K/Akt/mTORC1 axis is important as active mTORC1 plays a role in neuronal synaptic plasticity and in neuronal survival during embryonic development (Morita et al., 2015). Hormones and growth factors such as insulin, insulin-like growth factor (IGF-1) and epidermal growth factor trigger mTORC1 activity through a cascade of events that begins with the receptor-mediated activation of phosphatidylinositol 3-kinase-related kinase protein (P13K) through phosphorylation of the insulin receptor substrate (IRS1 and IRS2; Figure 1B; Um et al., 2006). mTORC1 rather than mTORC2 is regulated by nutrients such as amino acids (in particular but not exclusively, leucine) and glucose (Figure 1C; Gulati et al., 2008). Ultimately activation of the eukaryotic initiation factor 4E (eIF4E), its repressor eIF4E binding protein (4E-BP1), and p70S6K results in increased protein translation and synthesis, but lipid and nucleotide synthesis are also regulated (Goberdhan et al., 2016). Under fed conditions, mTORC1 regulates autophagy through phosphorylation at Ser757 of the ULK1 complex and blocks its interaction with 5′ AMP-activated protein kinase (AMPK), preventing autophagosome initiation (Long and Zierath, 2006). However, reports of inhibition at the maturation step through phosphorylation of UVRAG, extends its influence at several stages of autophagy (Liang et al., 2008). Conversely, low glucose, depletion of amino acids and oxidative stress are all key negative regulators of mTORC1 but stimulators of autophagy, where a balance between protein synthesis and clearance maintains cellular homeostasis (Wang et al., 1998). Sustained activation of p70S6K however, also phosphorylates IRS1 at inhibitory sites, negatively regulating Akt and stimulating autophagy (Shah et al., 2004). This fine line between stimulation and inhibition seems to decide direction and we question if there is scope to also consider a gray area, a period where these signaling metabolons form a synchronized collaboration between transitions. Pathways, which influence and respond to mTORC1 activity, such as the RAS-extracellular signal-regulated kinase (Ras-ERK), AMPK and mitogen-activated protein kinase (MAPK), expose a highway of networks that will be altered should mTORC1 activity change (Mendoza et al., 2011).

## Leucine A Dual Role in mTORC1 and Autophagy

Leucine was the first of the amino acids shown to activate mTORC1, blocking autophagy (Hara et al., 1998; Beugnet et al., 2003), but other amino acids such as glutamine, serine and arginine also function as key effectors (Jewell et al., 2015; Carroll et al., 2016). Although the mechanistic details are far less understood than the insulin/IGF pathway, their importance is gaining impetuous as the amino acid profiles or their metabolic enzymes are altered in several disease conditions, including Type 2 diabetes mellitus (T2DM) and AD (Vannini et al., 1982; Wang et al., 2011; Hull et al., 2015). The coupling of amino acid transport and metabolism is intrinsically linked, where activity of the system L (LAT) and system A transporter influence mTORC1 (reviewed in Dodd and Tee, 2012; Goberdhan et al., 2016). In brief, leucine is imported by the solute carrier family 7 member 5 (SLC7A5), which requires glutamine exchange through the Na^+^-linked system-A transporter (or system ASC (SLC1A5), coupled with the glycoprotein CD98 (Nicklin et al., 2009). Studies indicate that p70S6K was not activated until glutamine was exchanged for leucine, and reactivation of starved cells was dependent on glutamine uptake (Chen et al., 2014). Several factors are involved in amino acid signaling including but not limited to the Rag GTPases, the MAP4K3/GLK pathway, leucyl-tRNA synthetase, the adaptor protein p62 and P13K/hVps34 (Figure 1C; reviewed in Meijer et al., 2015).

Cellular uptake of leucine activates Rag GTPase heterodimers (RagA/B and RagC/D; Sancak et al., 2008; Sancak and Sabatini, 2009), which is dependent on hVps34 expression (Nobukuni et al., 2005). Activated Rag A/B-GTP binds mTORC1, through Raptor, and recruits mTORC1 via the Ragulator complex (MP1, p14 and p18) to the lysosome membrane, where Rheb resides (Kogan et al., 2010). The signaling adaptor p62, which influences cell survival and autophagy, has also been assigned a role in the amino acid induced recruitment of mTORC1 to lysosomes (Duran et al., 2011). Ultimately, Rheb a GTPase, now in close proximity, activates mTORC1-GTP and autophagy is inhibited through ULK1/2 and AMPK phosphorylation, increasing protein synthesis. Through amino acid signaling, a protein complex, called GATOR, and their regulators Sestrin 1/2 and CASTOR 1 modulate the interaction of Rags with mTORC1 (Chantranupong et al., 2014). Interestingly, hVps34, long associated with autophagy, shows increased expression in response to amino acids (Nobukuni et al., 2005). Activation of hVps34 by amino acids induced complex formation with hVps15, which is targeted to early endosomes by Rab7 supporting recruitment of proteins containing FYVE or PX domains (Um et al., 2006). Conversely, as described above during amino acid deprivation, hVps34 in a complex with Beclin 1, UVRAG and hVps15 drives autophagy. Thus, hVps34 expression is a shared protein between mTORC1 and autophagy regulation, the association of which seems to be dictated by nutrient load.

The mitochondrial protein, glutamate dehydrogenase (GDH), has been hypothesized to contribute to mTORC1 and autophagy regulation (Meijer and Codogno, 2008). GDH catalyzes the conversion of glutamate to α-keto glutarate (α-KG) releasing ammonia and NADH. It is thought that α-KG (potentially through propylhydrylase) activates RagB, driving mTORC1 (Durán et al., 2012). What is interesting is that the human branched chain aminotransferase (hBCAT) protein (hBCAT), which catalyzes the transamination of the branched-chain amino acids (BCAAs) and α-KG to glutamate and their respective α-keto acids (Conway and Hutson, 2016), have not been considered in these proposals. The hBCAT proteins are redox sensitive proteins (Conway et al., 2002, 2004, 2008), which form a metabolon with GDH in their reduced form, but when oxidized catalysis is reversibly inactivated (Islam et al., 2007). We hypothesize that in its reduced form hBCAT favors glutamate production through leucine transamination, important for GDH activity and thus mTORC1 activation. However, through either amino acid depletion or through an increase in oxidative stress this unique redox switch changes the function of hBCAT preventing metabolon formation with GDH reducing α-KG. This would inhibit mTORC1 activity and stimulate autophagy. Our work has shown that when hBCAT is overexpressed there is a significant increase in the level of p70S6K and a concomitant reduction in autophagy (unpublished observations). The dynamics and vectorality of these mechanisms are not entirely clear but more than likely will involve a nano-switch, such as that described for hBCAT, which responds to changes in cellular homeostasis.

## mTORC1, Autophagy and Alzheimer’s Disease

**Figure 2:**
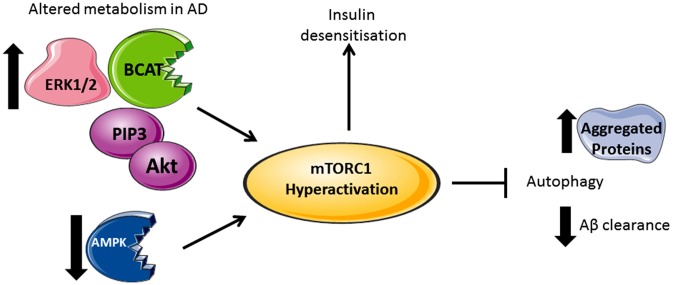
Divergent metabolic regulation in Alzheimer’s disease (AD). In AD brain, levels of Akt, PIP3, ERK1/2, and human branched chain aminotransferase (hBCAT) are increased together with phosphorylation of mTOR at Ser248, 4EBP1, eukaryotic initiation factor 4E (eIF4E) and p70S6K. The decreased expression of PTEN, an inhibitor of PI3K/Akt signaling, further activates mTOR. Over-activation of the PI3K/Akt/mTOR axis inhibits autophagy, supported by reduced levels of Beclin-1 and LC3 in the AD brain, thus reducing aggregate clearance including Aβ. Accumulative Aβ levels, alongside increased p70S6K, cause phosphorylation and inhibition of IRS1 instigating insulin desensitization. mTOR hyperactivation and the associated changes in metabolic proteins correlate with tau pathology and cognitive decline.

Under conditions where AD pathology persists, there is a reported loss of mTORC1 regulation, resulting in aggregate accumulation in the cell (Figure 2). Indeed, levels of Akt activation (Griffin et al., 2005), mTORC1 phosphorylated at Ser248 only, together with phosphorylated 4EBP1 (Li et al., 2005), p70S6K (Sun et al., 2014) and eIF4E (Li et al., 2005) were significantly increased in AD brain and correlated with Braak staging and tau pathology, indicating that protein translation is radically disordered. mTORC1 hyper-activation also correlated with cognitive decline in AD individuals (Caccamo et al., 2010; Sun et al., 2014). Neurons immunoreactive to PTEN show reduced expression in AD hippocampus and temporal cortex with a negative correlation to the severity of NFTs and plaques (Griffin et al., 2005). As PTEN attenuates PI3K/Akt signaling, through the dephosphorylation of PIP_3_, reduced levels can result in the hyper-activation of Akt signaling driving mTORC1 activity. Over activation of the PI3K/Akt/mTORC1 axis would inhibit autophagy and potentially contribute to reduced clearance of Aβ. However, we also need to consider that this would generate sustained p70S6K activation, which should phosphorylate IRS1, causing insulin desensitization and interrupted Akt activation, an apparent contradiction to what was reported in post-mortem AD brain. The most likely explanation is that other pathways, independent of insulin activation of mTORC1, may be responsible, such as (but not limited to) the ERK1/2 pathway, which incidentally was also found to be upregulated in AD brain and cell models (Young et al., 2009; Morales-Corraliza et al., 2016). In cell models, Aβ accumulation exacerbated mTORC1 signaling through phosphorylation of proline-rich Akt substrate of 40 kDa (PRAS40), promoting mTORC1 activity and inhibiting autophagy (Caccamo et al., 2011; Tramutola et al., 2015). Inhibition of mTORC1 using rapamycin alleviated Aβ accumulation and improved cognitive function in AD mice models, supporting this pathway as a future target to regulate neuronal health (Caccamo et al., 2010). However, upstream of mTORC1, we also find that disruptions to glucose and amino acid metabolism linked to insulin resistance and AD pathology add even further complexity.

## Insulin Resistance and Leucine Metabolism in Alzheimer’s Brain

Increasing evidence indicates that T2DM doubles the risk of developing AD as well as causing accelerated onset (Biessels et al., 2006; Domínguez et al., 2014; Exalto et al., 2014). Reports of insulin resistance and reduced expression of IGF1 and insulin receptors in AD brain has been linked to mild-cognitive impairment (Steen et al., 2005; Lu et al., 2013; Kim et al., 2015). Insulin resistance is considered to be further perpetuated by levels of Aβ oligomers through increased phosphorylation of the IRS1 inhibitory residue (Ser307; Moloney et al., 2010; O’Neill et al., 2012; Tramutola et al., 2015). Desensitization of neurons to insulin/IGF-1 responses will result in reduced glucose utilization and deficient energy metabolism (Mosconi, 2005). One would anticipate that mTORC1 activation through insulin would subsequently be lost and low cellular glucose should activate the AMPK pathway, further inhibiting mTORC1 driving autophagy. However, in addition to Aβ stimulation of mTORC1, AMPK activity was shown to be diminished in aged brain, and even more pronounced in T2DM, despite reduced intracellular glucose, which may also explain how hyperactivation of mTORC1 persists in the absence of insulin (Kodiha and Stochaj, 2011). Additionally, insulin degrading enzyme (IDE), which regulates extracellular Aβ degradation, showed reduced expression and activity that negatively correlated with Aβ levels in AD (Vekrellis et al., 2000; van der Heide et al., 2006; Zhao et al., 2007). Interestingly, enhanced IDE activity in APP double transgenic mice reduced Aβ levels in the brain preventing plaque formation (Leissring et al., 2003). Together these studies suggest that upregulation of IDE offers therapeutic benefits to target Aβ plaque removal *in vivo*. Intra-hippocampal administration of insulin in a T2DM rat model attenuated cognitive impairment (McNay et al., 2010) and in a separate study nasal insulin administration in a diabetic mouse model improved diabetic-related decline in cognitive function, offering evidence that overcoming insulin resistance may have therapeutic benefits in AD patients (Wang et al., 2010). A pilot study in humans showed that treatment with intranasal insulin improved delayed memory and preserved general cognition (Craft et al., 2012). However, larger more in-depth studies will decide if this treatment has sustained impact overtime.

Increased blood levels of BCAAs positively correlate with insulin resistance and have been used as signature profiles for T2DM, insulin-resistant states of obesity and Huntington’s disease (Vannini et al., 1982; Mochel et al., 2007, 2011; Wang et al., 2011). For those individuals with T2DM-associated AD increased BCAAs, particularly leucine, could over activate mTORC1 signaling through the various pathways highlighted. Hyperleucinamia in a T2DM mouse model showed that retromer trafficking was impaired, with decreased levels of hVps34 reported, whereby hyperleucinemia may account in part for insulin resistance and driving mTORC1 activation independent of insulin (Morabito et al., 2014). Our group has reported that the hBCAT proteins are significantly upregulated in AD brain relative to age-matched controls and in dementia with lewy bodies and vascular dementia (Hull et al., 2015; Ashby et al., 2017). Here, we proposed that an increase in these proteins was initially to protect neuronal cells through glutamate regulation. In light of our recent work, we now extend their role in brain metabolism as indirect modulators of mTORC1 and autophagy. Here, we suggest that overexpression of hBCAT could contribute to the hyperactivation of mTORC1, disrupting autophagy, potentially through GDH metabolism of glutamate.

On the other hand, supplementation of BCAAs improved glucose homeostasis and insulin resistance in patients with hepatic cirrhosis (Kawaguchi et al., 2008) and increasing dietary leucine intake improved glucose and cholesterol metabolism in mice, indicating that a balance must be met to avoid disequilibrium (Zhang et al., 2007). Contrary to T2DM, levels of BCAAs were reduced in patients recovering from traumatic brain injury and supplementation contributed to improved cognitive function, observed both in humans and rat models of TBI (Vuille-Dit-Bille et al., 2012; Jeter et al., 2013). Although the exact mechanisms controlling this balance between nutrient load and pathology remains elusive these studies highlight the potential for diet to significantly impact these regulatory pathways and should we get the balance correct may be able to delay the onset of AD.

## Conclusion

This review has primarily focussed on dysregulated nutrient signaling that impacts autophagy at early endosome formation offering insight into potential pathways that are dysregulated in AD. Clearly, our understanding of how mTORC1 and these signaling networks regulate protein aggregation is far from complete. Importantly, nutrients and growth factors control these pathways and we potentially have an opportunity to regulate brain metabolism through diet. This may be important in neurodegenerative conditions as levels of amino acids are significantly increased in HD and T2DM. However, dysfunctional retromer-dependent trafficking will also be key, in particular with respect to its regulation by nutrient load and cellular stress. It is likely that these pathways operate as metabolons, where clearly a change in function for key metabolic proteins is important for regulation. We speculate that this change in function is triggered by changes in cellular homeostasis, governed by nutrient signals, hypoxia or hormones, and an understanding of which could identify key targets for future neurodegenerative therapeutics. Targeting autophagy and its regulation is therefore of value, where an understanding as to how this mechanism can be controlled will be important to maintain neuronal health.

## Author Contributions

All authors contributed to the content of the article.

## Conflict of Interest Statement

The authors declare that the research was conducted in the absence of any commercial or financial relationships that could be construed as a potential conflict of interest.
